# A Rare Culprit or an Elusive Culprit in Disguise? Unraveling Wild-Type ATTR Cardiac Amyloidosis in Heart Failure With Reduced Ejection Fraction

**DOI:** 10.1177/23247096251345712

**Published:** 2025-05-30

**Authors:** Jose Loayza Pintado, Taiwo Ajani, Daniela Hernandez, Everardo Cobos

**Affiliations:** 1 University of Texas Rio Grande Valley School of Medicine, Edinburg, USA

**Keywords:** cardiac amyloidosis, systemic amyloidosis, transthyretin amyloidosis, restrictive cardiomyopathy, heart failure

## Abstract

Cardiac amyloidosis (CA) is a rare disorder caused by the deposition of abnormal proteins called amyloid in the myocardium, leading to dysfunction. The 2 most common forms of amyloidosis are AL (light chain) and ATTR (transthyretin). Diagnosing amyloidosis is challenging, especially in its early stages, due to its nonspecific symptoms and overlap with other conditions. Recent studies suggest that the incidence of wild-type transthyretin amyloidosis is rising, likely due to improved diagnostic techniques and an aging population. We present the case of a 72-year-old male with lower extremity edema, progressive shortness of breath, and worsening renal function. He had a significant medical history, including hypertension, small lymphocytic lymphoma, coronary artery disease, diabetes, and chronic kidney disease. Physical examination revealed orthostatic hypotension and peripheral neuropathy. Imaging showed restrictive cardiomyopathy with reduced ejection fraction. Laboratory tests confirmed anemia and proteinuria, while a bone marrow biopsy ruled out AL amyloidosis. A Tc-99m pyrophosphate scan confirmed the diagnosis of ATTR CA. ATTR often presents with multi-organ involvement, complicating diagnosis. This patient’s coexisting conditions, including orthostatic hypotension and renal failure, may have been aggravated by amyloidosis. Misdiagnosis between AL and ATTR can lead to inappropriate treatments, making accurate diagnosis crucial. ATTR requires transthyretin stabilizers and symptom management, while AL needs chemotherapy. Treatment of amyloidosis must be individualized, as autonomic dysfunction, arrhythmias, and renal involvement require careful management. Early diagnosis and differentiation are essential for appropriate treatment and improved outcomes in patients with multi-organ involvement.

## Introduction

Amyloidosis is a rare condition characterized by the accumulation of abnormal proteins, called amyloids, in various organs and tissues, disrupting their normal function. This buildup can affect organs such as the heart, kidneys, liver, and nervous system, leading to symptoms like fatigue, swelling, and organ failure.^1^ Cardiac amyloidosis (CA) involves the accumulation of amyloid fibrils in the extracellular matrix of the myocardium. The disease can be caused by different types of amyloid proteins, with the most common being AL (light chain) amyloidosis and ATTR (transthyretin) amyloidosis.^2^

However, amyloidosis is often missed due to its nonspecific symptoms, rarity, and overlap with other more common diseases.^3,4^ While improved awareness, earlier suspicion, and advances in diagnostic technologies have increased detection rates, diagnosing the condition—especially in its early stages—remains challenging.^5^ This is due to the disease’s varied clinical presentations, low prevalence, and sometimes ambiguous test results.^6^

Recent studies suggest that the incidence and prevalence of amyloidosis may be higher than previously thought, particularly for certain subtypes. This increase can be linked to advancements in diagnostic methods, particularly the use of technetium 99m-pyrophosphate myocardial imaging, which has shown high sensitivity in diagnosing ATTR CA.^7^ In general, the incidence of wild-type transthyretin (ATTRwt) amyloidosis rose from less than 3% of all cases between 1987 and 2009 to 14% during 2010 to 2015, and further to 25% in more recent years.^8^

These trends may be attributed to improved diagnostic techniques, greater awareness among healthcare providers, and an aging population, as amyloidosis often manifests in older individuals.^9^

Here, we present the case of an elderly male diagnosed with ATTRwt CA, highlighting the therapeutic complexities, particularly when it coexists with multiple seemingly unrelated co-morbidities, nonspecific symptoms, and a concomitant hematologic malignancy.

## Case Presentation

A 72-year-old male presented to the clinic with complaints of lower extremity edema and 2 previous hospital admissions due to newly diagnosed decompensated congestive heart failure (CHF) in the past 5 months. He reported progressive shortness of breath, exertional dyspnea, near-syncopal episodes, worsening renal function, and anemia over the preceding weeks.

The patient had a significant medical history, including hypertension, untreated small lymphocytic lymphoma (SLL), coronary artery disease with 2 prior cardiac stents, CHF with reduced ejection fraction (EF), diabetes mellitus, chronic kidney disease (CKD) Stage 3, anemia, degenerative joint disease with bilateral hip replacements at age 49, and lumbar spinal stenosis treated with L4-5 fusion.

The patient has a relevant family history, with an uncle diagnosed with CA and currently receiving treatment with Tafamidis, and an aunt who passed away due to complications related to CA.

On physical examination, the patient’s blood pressure measured 160/70 mmHg in the supine position and 125/50 mmHg when standing. Heart sounds were normal without murmurs, and his lungs were clear to auscultation. Bilateral 2+ pitting edema was noted in both ankles, while the remainder of the physical examination was unremarkable. He had a grade 1 to 2 peripheral neuropathy.

Laboratory results were notable for an elevated proBNP level of 10 249 pg/mL, a hemoglobin of 8.9 g/dL, a platelet count of 191 000/μL, an absolute neutrophil count of 4.38 × 10⁹/L, and an absolute lymphocyte count of 2.55 × 10⁹/L. The patient also had elevated creatinine (2.32 mg/dL) and significant proteinuria, with a urine protein-to-creatinine ratio of 6872. Flow cytometry revealed an immunophenotype consistent with chronic lymphocytic leukemia (CLL)/SLL, characterized by monoclonal B-cell proliferation. Serum protein electrophoresis (SPEP) and urine protein electrophoresis (UPEP) showed no monoclonal protein, with a kappa/lambda ratio of 1.34.

Bone marrow biopsy findings were consistent with CLL/SLL, with less than 1% plasma cells demonstrating a polytypic phenotype. Cytoplasmic light chain evaluation was normal, no amyloid deposits were noted on the specimen, and cytogenetics were normal. These findings ruled out light chain amyloidosis as a complication of the patient’s hematologic malignancy.

**Image 1.**
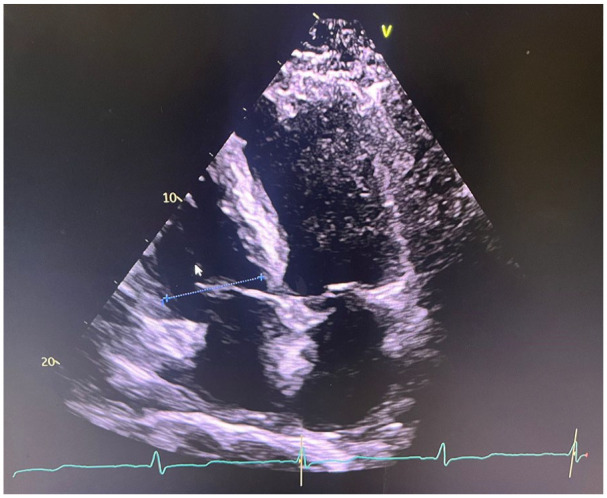
Apical 4-chamber echocardiogram view showing moderate left ventricular wall thickening with a granular, speckled myocardium.

An echocardiogram demonstrated an EF of 35%, features of restrictive cardiomyopathy, moderate left ventricular hypertrophy, severe left atrial dilation, a speckled myocardial pattern, and decreased global longitudinal strain with apical sparing, findings highly suggestive of CA. A Lexiscan Cardiolite scan showed an EF of 37% at rest, with minimal ischemia and mild apical defects, corroborating the cardiomyopathy findings (Image 1).

**Image 2.**
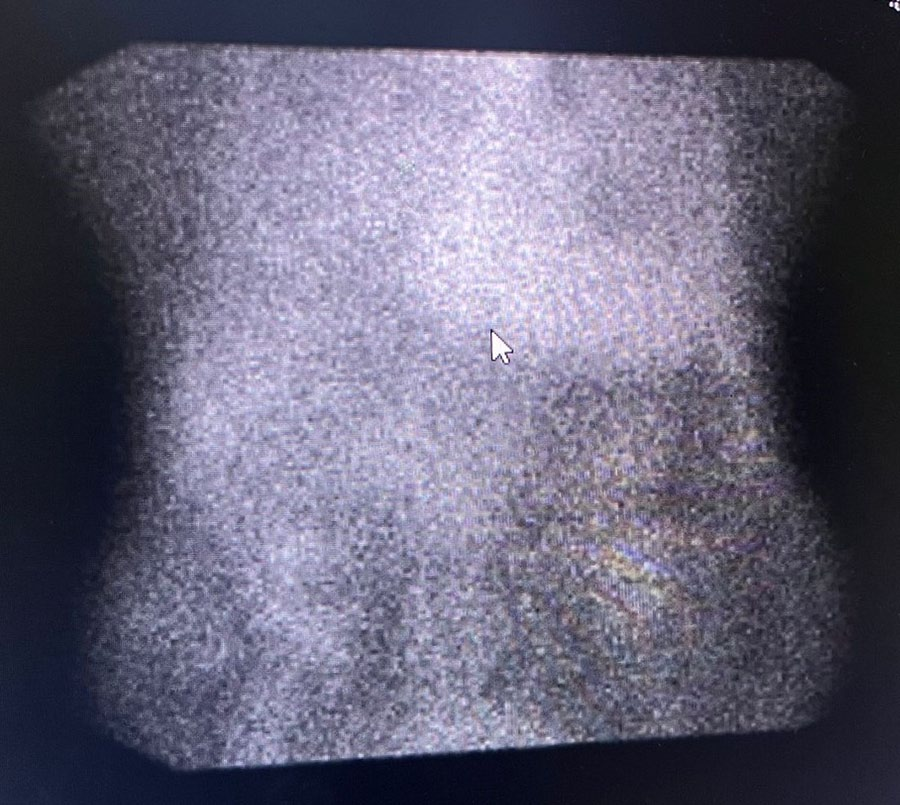
Tc-99m PYP scan showing extensive radiotracer accumulation throughout the cardiac region, demonstrating significant myocardial tracer uptake with a myocardial-to-lung ratio of ≥1.5. Tc-99m PYP, technetium-99m pyrophosphate.

Given the strong suspicion of CA, a technetium-99m pyrophosphate (Tc-99m PYP) scan was performed for ATTR amyloid, revealing intense myocardial tracer uptake with a myocardial-to-lung ratio ≥1.5. This finding confirmed ATTR CA. Genetic testing for hereditary ATTR was negative, with no mutations identified. The patient was initiated on Tafamidis following approval by the insurance provider (Image 2).

## Discussion

ATTR is a systemic condition that affects multiple organ systems.^1^ This patient had several comorbidities commonly associated with ATTR, in addition to cardiac failure. These included orthostatic hypotension, renal failure, hip and lumbar degeneration, diabetes, neuropathy, and anemia. The presence of a hematologic malignancy further complicated the clinical picture, underscoring highlighting the necessity of an accurate diagnosis.

ATTR frequently results in autonomic dysfunction, impairing blood pressure regulation and causing orthostatic hypotension, a common manifestation in patients with cardiac or neuropathic involvement.^10^

While not exclusive to ATTR, joint degeneration and orthopedic issues are often observed in these patients. Amyloid deposits have been found in musculoskeletal tissues, including tendons, ligaments, and joints. Additionally, ATTR can cause carpal tunnel syndrome and spinal stenosis, reflecting a broader musculoskeletal burden due to amyloid deposition.^11,12^

Peripheral neuropathy is a hallmark of ATTR, involving sensory, motor, and autonomic dysfunction. Neuropathy results from amyloid deposits affecting peripheral nerves, leading to symptoms such as pain, paresthesia, and weakness.^13^

Though amyloidosis itself does not directly cause diabetes, amyloid deposits in the pancreas or other organs can worsen existing diabetes or lead to secondary diabetes, complicating blood sugar management.^14^

Renal involvement, though more prevalent in light chain (AL) amyloidosis, also occurs in ATTR. Amyloid deposits in the kidneys impair glomerular function, leading to proteinuria and CKD. However, renal dysfunction in ATTR tends to be less severe than in AL.^15^

Cardiac involvement is a defining feature of ATTR, particularly the transthyretin subtype. Amyloid deposits in the myocardium lead to restrictive cardiomyopathy, manifesting as CHF features such as dyspnea, edema, and reduced EF. ATTR causes diastolic dysfunction and atrial dilation, which contributes to heart failure symptoms.^16^

Anemia in ATTR is likely multifactorial, resulting from chronic disease, renal dysfunction, and potential nutritional issues.^17^ Although not a hallmark feature of ATTR, the presence of anemia underscores the systemic nature of the disease, highlighting the need for a comprehensive evaluation.

Given the patient’s history of SLL, ruling out AL was essential. AL and ATTR are distinct in causes, treatments, and prognoses. AL is caused by misfolded light chains produced by clonal plasma cells or B-cell malignancies and is often associated with plasma cell dyscrasias like multiple myeloma.^18^ In contrast, ATTR results from misfolded transthyretin proteins, either from hereditary mutations or wild-type forms, and is unrelated to hematologic malignancies.^4^

Accurately diagnosis is critical, as AL and ATTR require entirely different treatment approaches. AL amyloidosis requires aggressive therapy targeting the underlying plasma cell disorder, such as chemotherapy or stem cell transplantation, as untreated AL amyloidosis progresses rapidly and has poor prognosis.^18^ On the other hand, ATTR is managed with Tafamidis, a transthyretin tetramer stabilizer. It inhibits the rate-limiting step in TTR amyloidogenesis, specifically tetramer dissociation. Clinical trials have demonstrated that Tafamidis significantly reduces mortality and cardiovascular-related hospitalizations compared to placebo.^19^ Other agents include transthyretin reduction therapies such as patisiran or inotersen, alongside symptomatic treatments for heart failure, such as diuretics. Chemotherapy, though critical for AL, would be ineffective and potentially harmful in ATTR.^20^

The prognosis of ATTR and AL differs significantly from each other. AL progresses more aggressively, often involving multiple organ systems such as the kidneys, heart, liver, and peripheral nerves, with a median survival of 6 to 12 months in cases with cardiac involvement if untreated.^18^ In contrast, ATTR, especially the wild-type form, tends to progress more slowly, and with appropriate management, patients can achieve longer survival and a better quality of life.^21^

Diagnostic tests used to rule out AL in this case included SPEP and UPEP with immunofixation, which showed no monoclonal proteins and a normal free light chain ratio, ruling out clonal plasma cell activity. Bone marrow biopsy revealed no amyloid deposits or plasma cell dyscrasia, further excluding AL in this patient with CLL/SLL. Additionally, a positive Tc-99m PYP scan confirmed ATTR CA, clarifying the diagnosis and ensuring the patient received appropriate treatment rather than unnecessary therapies for AL amyloidosis. While biopsy remains the gold standard for diagnosing amyloidosis, the Tc-99m PYP scan, a non-invasive method, proved sufficient for confirming ATTR CA. PYP scans have shown high sensitivity and specificity for detecting CA, particularly ATTR, and are increasingly used in clinical practice as an alternative to biopsy.^22,23^

Managing blood pressure and cardiac function in amyloidosis is complicated by organ dysfunction, autonomic abnormalities, and the risks posed by conventional treatments. Many patients, particularly those with cardiac involvement, experience orthostatic hypotension and autonomic dysfunction due to amyloid deposits affecting the heart and nervous system.^10^ This impairs the body’s ability to regulate blood pressure, complicating the use of traditional antihypertensive treatments.

Diuretics are commonly used to manage fluid retention in CA^16^ but must be carefully balanced to avoid exacerbating hypotension, kidney dysfunction, or electrolyte imbalances. Beta-blockers, while standard in heart failure, may be less effective in amyloidosis^24^ due to the restrictive nature of the heart and can worsen low blood pressure or orthostatic hypotension.

Arrhythmias, including atrial fibrillation and ventricular arrhythmias, are frequent in amyloidosis due to amyloid deposits affecting the heart’s conduction system.^16^ Managing these arrhythmias is challenging due to the altered electrical properties of the heart. Structural changes, including atrial dilation and impaired electromechanical function, increase the likelihood of thrombus formation, even in the absence of atrial fibrillation. Studies show that up to 16% of these patients experience thromboembolic events. Anticoagulation therapy is often recommended, especially when the risk of bleeding is manageable.^25^

Due to the limited evidence base and the need for more targeted treatments for amyloidosis-related cardiac issues, tailored, individualized approaches are crucial. Ongoing research is critical to refine management strategies and address the challenges of treating this complex condition.

In summary, the patient’s symptoms, physical findings, laboratory results, and cardiac imaging were consistent with acquired transthyretin CA. The combination of these findings, particularly in an older adult with systemic symptoms and cardiac abnormalities, strongly suggests ATTR, emphasizing the need for thorough evaluation, as demonstrated in this case.

## Conclusion

This case underscores the importance of a thorough diagnostic workup for patients with heart failure, particularly in older individuals with multi-organ dysfunction and systemic symptoms. Early identification and differentiation of amyloidosis subtypes are critical for appropriate management, as treatment strategies for AL and ATTR CA diverge significantly. Managing the complex symptoms of ATTR CA, especially when accompanied by other amyloidosis-related complications, demands an individualized approach, highlighting the need for ongoing research to refine management strategies.
